# Temozolomide in Combination With NF-κB Inhibitor Significantly Disrupts the Glioblastoma Multiforme Spheroid Formation

**DOI:** 10.1109/OJEMB.2019.2962801

**Published:** 2020-02-17

**Authors:** Hui Xia, Naze G. Avci, Yasemin Akay, Yoshua Esquenazi, Lisa H. Schmitt, Nitin Tandon, Jay-Jiguang Zhu, Metin Akay

**Affiliations:** ^1^ Biomedical Engineering DepartmentUniversity of Houston 14743 Houston TX 77204 USA; ^2^ Mischer Neuroscience Associates and the Vivian L. Smith Department of NeurosurgeryUniversity of Texas Health Science Center in Houston, UTHealth and Memorial Hermann 220841 Houston TX 77030 USA; ^3^ Biomedical Engineering DepartmentUniversity of Houston 14743 Houston TX 77204 USA

**Keywords:** Drug-screening, glioblastoma, microfluidic, PEGDA, 3D cell culture

## Abstract

Glioblastoma multiforme (GBM) is the most common malignant primary brain tumor, accounting for 50% of all cases. GBM patients have a five-year survival rate of merely 5.6% and a median overall survival of 14.6 months with the “Stupp” regimen, 20.9 months with tumor treatment fields (TTF, OptuneR) in patients who participated in clinical trials, and 11 months for all GBM patients prior to TTF use. *Objective:* Our group recently developed a brain cancer chip which generates tumor spheroids, and provides large-scale assessments on the response of tumor cells to various concentrations and combinations of drugs. This platform could optimize the use of tumor samples derived from GBM patients to provide valuable insight on the tumor growth and responses to drug therapies. To minimize any sample loss *in vitro*, we improved our brain cancer chip system by adding an additional laminar flow distribution layer, which reduces sample loss during cell seeding and prevents spheroids from escaping from the microwells. *Methods:* In this study, we cultured 3D spheroids from GBM cell lines and patient-derived GBM cells *in vitro*, and investigated the effect of the combination of Temozolomide and nuclear factor-κB inhibitor on tumor growth. *Results:* Our study revealed that these drugs have synergistic effects in inhibiting spheroid formation when used in combination. *Conclusions:* These results suggest that the brain cancer chip enables large-scale, inexpensive and sample-effective drug screening to 3D cancer tumors *in vitro*, and could be applied to related tissue engineering drug screening studies.

## I. Introduction

Glioblastoma multiforme (GBM) is the most common and aggressive type of primary malignant brain tumor, and accounts for ∼50% of all cases. Annual incidence of GBM is 3.21 per 100,000 population; 13,310 patients are expected to be diagnosed in 2019 [1]. GBM has a poor prognosis, with a five-year survival rate of merely 5.6% [1] and median overall survival of 14.6 months with the “Stupp” regimen (radiation with daily temozolomide (TMZ) x 4-6 weeks followed by cyclic TMZ) or 20.9 months with the “Stupp” regimen plus tumor treating fields (TTF, OptuneR) [2], [3] in patients who participated clinical trials [4]–[6]. However, variable responses during chemoradiation and developing resistance to chemotherapy drugs including TMZ, are the major challenges to clinically treat GBM. Contributing factors that lead to chemoradiation resistance include tumor genetic diversity and complex heterogeneity, as well as expression of DNA repair enzymes [7]. Previous studies have demonstrated that the overexpression of O6-methylguanine-DNA methyltransferase (MGMT), which repairs the TMZ-induced guanine damage in DNA, is one of the major factors for chemoresistance to TMZ in GBM cells [8], [9]. Therefore, there is an urgent need for an efficient approach to identify new drug treatment regimens that maximize tumor cell lysis and reduce subsequent drug resistance.

Nuclear factor-κB (NF-κB) protein is a regulatory transcription factor consisting of a family of structurally-similar transcription factors: p65 (RelA), RelB, c-Rel, p105/p50, and p100/p52 that form homo- and heterodimer to regulate transcription of target genes. NF-κB regulates various signal transduction pathways that are associated with cell survival, proliferation, migration, angiogenesis and apoptosis [10]–[14]. NF-κB also promotes chemoresistance to TMZ alkylating agent and plays an important role in the regulation of MGMT activity in GBM by activating MGMT gene expression through NF-κB binding sites within the MGMT promoter [15]. Inhibition of NF-κB activity by sulfasalazine, an anti-inflammatory drug, induced cell apoptosis in GBM cell lines [16]. Another study showed that inhibiting the nuclear translocation of NF-κB by using a decoy oligonucleotide resulted in a significant reduction in cell numbers up to 45% compared to control [17]. The inhibition of endogenous NF-κB activity in patient-derived GBM stem cells (GSCs) culture, using the selective IKKβ antagonist, or siRNA-mediated knockdown of IKKβ and/or RelA, has been shown to significantly decrease tumor sphere formation, suggesting that NF-κB is involved in the self-renewal capacity of GSCs [18]. Additionally, parthenolide inhibits NF-κB activity via inhibiting IKβ kinase and by modifying p65 at a key cysteine residue in its activation loop [19], and has been shown to reduce Akt phosphorylation and activate mitochondrial apoptosis signaling in GBM cells [20]. These results demonstrate that parthenolide exhibits anti-tumor effects of GBM cells through inhibition of NF-κB and Akt signaling, as well as the activation of apoptotic proteins [20]. Furthermore, NF-κB inhibitor BAY 11-7082 suppressed the expression of MGMT in TMZ resistant U251 GBM cell line and enhanced TMZ-induced cytotoxicity and apoptosis, further suggesting that the NF-κB pathway and MGMT interact to promote TMZ resistance [21].

To understand infiltrative and aggressive progression of GBM, it is important to develop a tumor model which resembles in vivo milieu. *In vitro* models based on two-dimensional (2D) monolayer culture are frequently used for cancer studies including tumor growth, proliferation and invasion [22]. However, the 2D cell culture model fail to demonstrate natural cell-to-cell and cell-to-extracellular matrix (ECM) interactions in vivo [23], [24]. Therefore, scaffolds and microstructures which enable effective three-dimensional (3D) *in vitro* GBM models that can recapitulate in vivo features of the tumor have been investigated [25]–[27]. However, a major drawback of these reported scaffolds and microstructures is their limited application for drug screening. The lack of a suitable drug distribution system in these reports makes simultaneous drug tests with various combinations and concentrations difficult to perform. To address this challenge, our group previously reported a microfluidic device, which integrated a microwell array with tree-shaped microfluidic feeding channels. These channels enable simultaneous testing of a range of concentrations of two different drugs, distributed among the microwells [28], [29]. This low-cost and easy to produce platform enables spheroid culturing in a relevant 3D model, simulates the in vivo brain microenvironment, and allows the testing of different drug combinations to potentially determine the appropriate drug treatment regimen for a specific patient.

Using this system, we recently reported that the combination of TMZ with bevacizumab (BEV), which is currently used for the treatment of recurrent GBM, contributed to increased tumor cell death compared to individual drug treatment in patient-derived 3D GBM spheroids [28]. This study suggested that cells derived from different patients respond differently to the drug treatment, in agreement with other reports [28], [30], [31]. The mechanisms of various responses to the same drug regimens are complex, and are likely in part due to the heterogeneity of the tumor, including the presence of cancer stem cells. This heterogeneity is the main limitation of effective GBM treatment in patients. Therefore, in this study, we used our 3D *in vitro* brain cancer chip platform to further investigate GBM-specific drug treatment effects by examining the responses of multiple drug combinations. To this aim, we measured the co-effect of inhibiting the transcription factor NF-κB combined with TMZ in human GBM spheroids derived from cell lines (U87 and LN229) [32], as well as in patient-derived GBM spheroids. We found that using NF-κB inhibitor BAY 11-7082 alone decreased tumor viability *in vitro* for both GBM sources, i.e., cell lines and patient-derived cells. However, it should be noted that the combined effect of BAY 11-7082 and TMZ granted a stronger reduction of tumor cell viability, tumor growth, and ultimately caused the destruction of 3D tumor spheroids.

## II. Results

### A. Improvement of Brain Cancer Chip Devices

**Figure 1.**
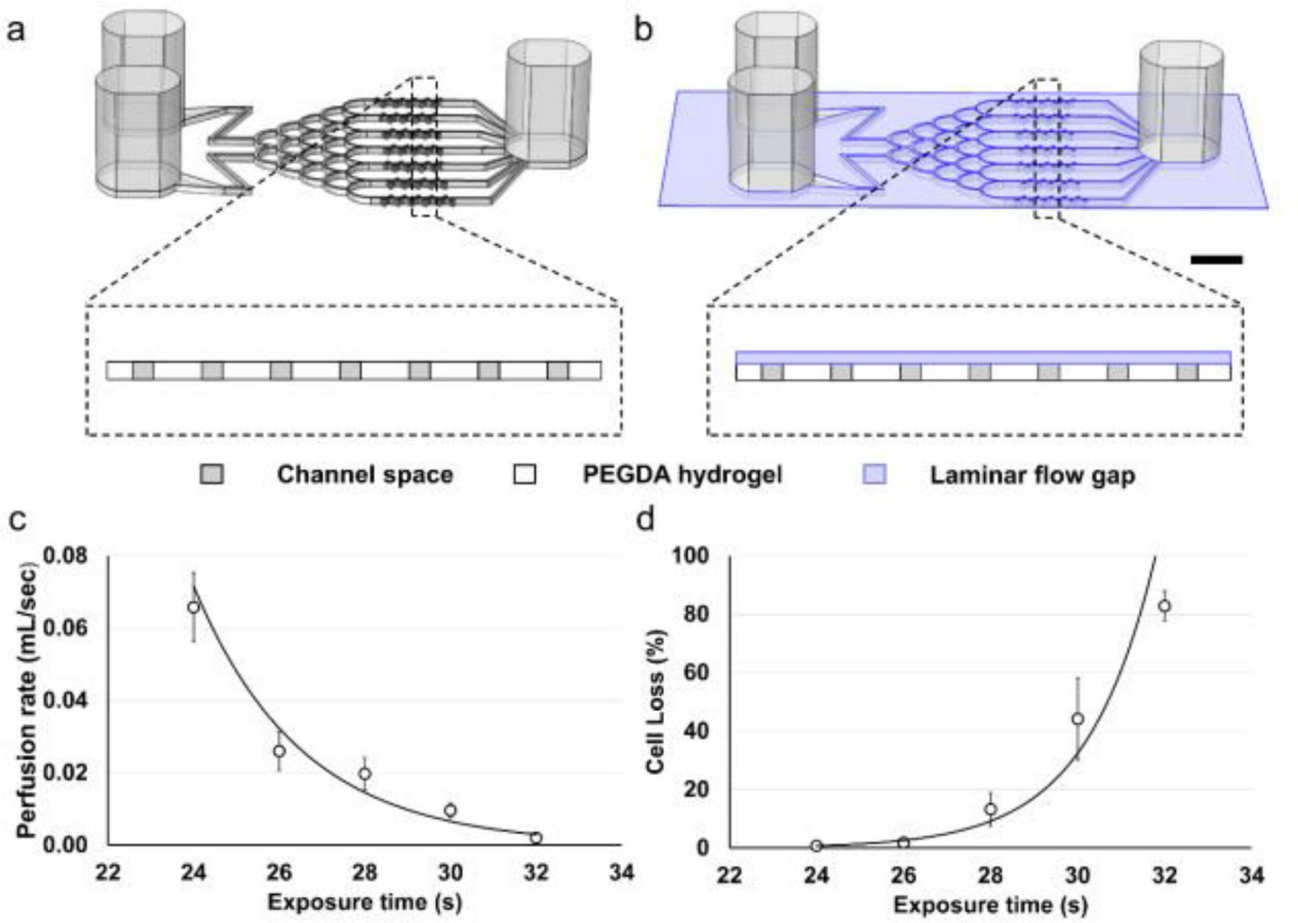
Schematic and optimization of the brain cancer chip and microfluidics application using a polyethylene glycol diacrylate (PEGDA) hydrogel. (a) Schematic of the original brain cancer chip design, without the laminar flow layer, and a cross section over the microwell array. (b) Schematic of the brain cancer chip design with the additional laminar flow layer, and a cross section over the microwell array. Scale bar is 5 mm. (c) Perfusion decreases as exposure time increases (n = 4). (d) Cell loss increases during the cell seeding stage as exposure time increases (n = 4).

The design of the brain cancer chip has been previously described in detail [28]. We implemented a photolithography-based approach to enable rapid fabrication of a hydrogel-based microfluidic device for culturing 3D brain cancer spheroids *in vitro*. This approach does not rely on the use of cleanroom environment or costly and labor intensive fabrication processes such as photoresist-spinning, replica molding, and plasma bonding, which are essentials in conventional polydimethylsiloxane (PDMS)-based microfluidic devices [28], [29]. The original brain cancer chip design is comprised of three reservoirs (two inlets and one outlet) connected by seven microfluidic channels, each containing a series of dead-end microwells, that rely on the initial momentum to seed the cells (Fig. 1(a)) [28]. In practice, the majority of the cells perfused through the inlet could be lost to the outlet, rather than establishing culture in one of the microwells. We eliminated this limitation by adding a thin laminar flow distribution layer between the top glass slide and the hydrogel body (Fig. 1(b)). This layer is deep enough to allow single cells to move in a laminar manner during the cell seeding stage. However, once the spheroids are formed in the microwells, the shallow depth of the layer prevents spheroid loss from the microwells.

The laminar flow distribution layer was created by exploiting the UV-scattering feature of polyethylene glycol diacrylate (PEGDA). While the aqueous solution of 20% PEGDA (700 MW) is transparent, the polymerized PEGDA hydrogel uniformly scatters the incoming UV [33], [34]. Thus, when the PEGDA solution is sandwiched between two glass slides and is exposed to UV light, the upper and the lower layers of PEGDA do not crosslink simultaneously. When the top layer crosslinks, it becomes opaque and scatters the incoming light. As a result, this top layer prevents the crosslinking of the lower layer. By controlling the UV exposure time, the amount of crosslinked hydrogel can be fine-tuned to fabricate a brain chip with a gap of certain depth between the top glass side and the hydrogel body.

We optimized the depth of the laminar flow layer and the seeding cell loss by controlling the exposure time of the chip to UV light. The depth of the laminar flow layer was derived from the flow resistance of the chip, which was obtained by measuring the flow perfusion rate when the chip was tilted at 45 degrees and the inlets are higher than the outlet. Fig. 1(c) illustrates that when the chip was exposed for 32 s (equivalent to 640 mJ/cm^2^), the perfusion rate of the chip was 0.12 ± 0.01 mL/min, and the estimated depth of the laminar flow layer was nearly zero (i.e., the PEGDA hydrogel achieved full crosslinkage and subsequently reduced flow). Using lower exposure times of 30, 28, 26, and 24 s, the perfusion rate of the chip increased to 0.58 ± 0.12, 1.18 ± 0.27, 1.56 ± 0.32, and 3.95 ± 0.57 mL/min, and the depth of the laminar flow gap was estimated to be 57.3, 92.6, 106.6, and 162.1 µm, respectively. Fig. 1(d) demonstrates the brain cancer chip's cell loss during the seeding stage over various UV exposure time. When the chip was exposed for 24, 26, or 28 s, the cell loss remained as low as 0.8 ± 0.2%, 1.8 ± 0.6%, and 13.2 ± 5.6%, respectively. However, using higher UV exposure time of 30 and 32 s, the cell loss increased to 44.2 ± 14.0% and 82.8 ± 5.1%, respectively. Additionally, to prevent spheroids from being washed out of the microwells, the exposure time has to be long enough to keep the depth of the laminar flow gap small, while it also needs to be short enough to let single GBM cells enter. Thus, we proceeded to fabricate the brain cancer chip using the exposure time of 28 s (estimated gap size of 92.6 µm) to seed and culture GBM cell spheroids. Considering that a diameter of the GBM cell is approximately 15 µm, the laminar flow layer under such depth would prevent any grown spheroid of diameter larger than 6 cells from being washed out of the microwells.

### B. Effect of NF-κB Inhibitor and TMZ on 3D GBM Spheroids Formation

**Figure 2.**
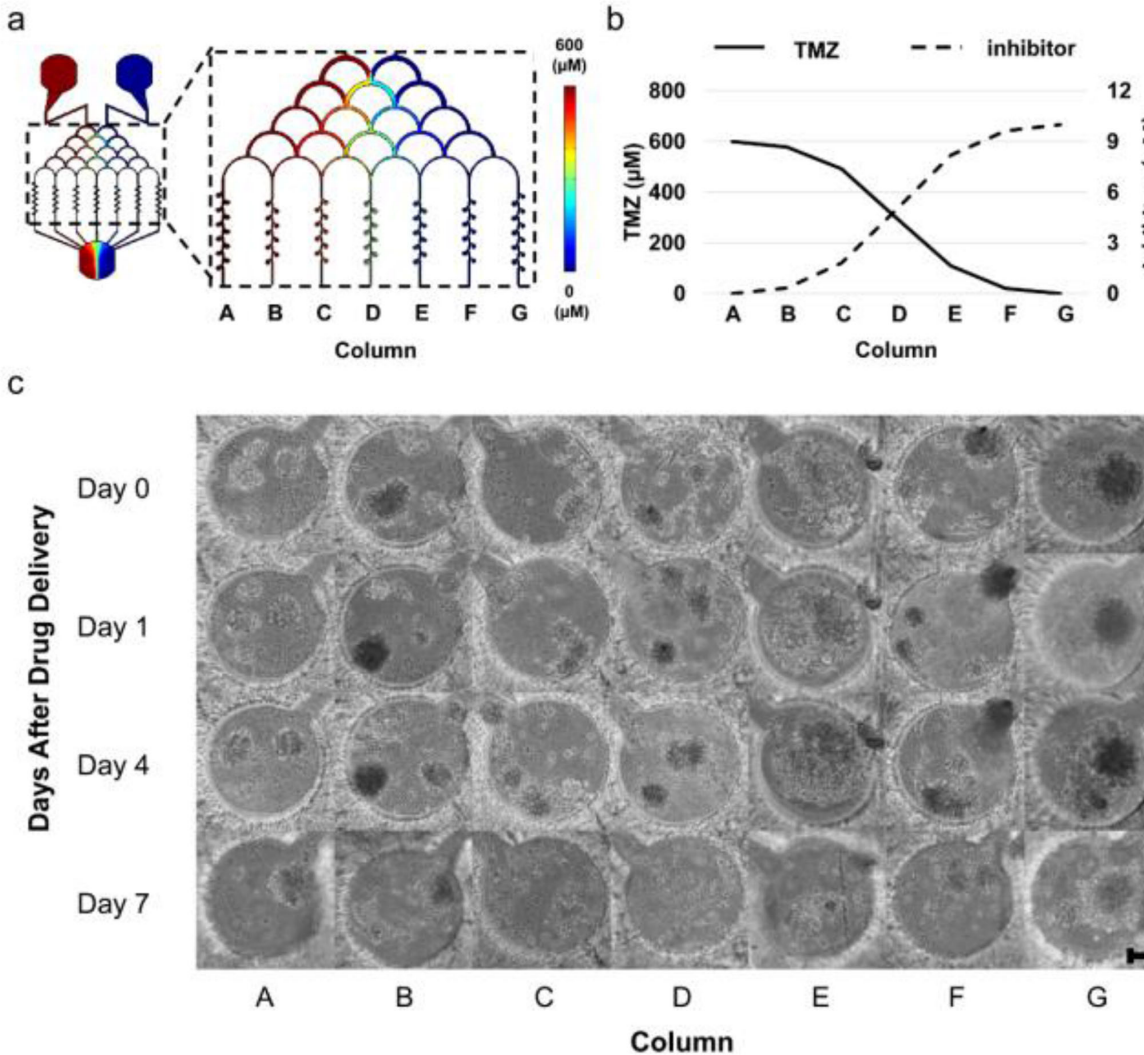
Drug concentration distribution in the laminar flow layer enhanced brain chip. (a) Drug concentration distribution in the laminar flow layer enhanced brain cancer chip, simulated for the TMZ side (left). (b) Simulation-derived diagram showing drug concentration in each of the columns in the laminar flow layer enhanced brain chip. Straight and dashed lines represent the concentration of TMZ and BAY 11-7082, respectively. (c) Representative images of LN229 spheroids over 7 days of spheroid culture after the drug administration. TMZ (600 μM, left inlet) and BAY 11-7082 (10 μM, right inlet) were applied to LN229 spheroids in the brain cancer chip. Effects of the drug treatment on the GBM spheroids were visualized by the spheroid collapsing and the disaggregation of dead cells from the spheroids on day 4-7. Scale bar is 100 μm.

Using a finite element simulation, we calculated the distribution and approximate concentration of NF-κB inhibitor BAY 11-7082 and TMZ in microwells from each microchannel (Fig. 2(a)). After drug injection, the concentration of TMZ decreased to 99.9%, 96.4%, 82.0%, 49.8%, 18.2%, 3.3%, and 0.1% of the initial inlet concentration in the microwells at column A-G, respectively (Fig. 2(b)). Similar column-based concentration decreases also happened for BAY 11-7082. Thus, a combined concentration gradient between BAY 11-7082 and TMZ was formed. To understand the inhibitory effect of this combined drug gradient on GBM cell proliferation, we used three different GBM cells types, i.e., LN229, U87 and patient-derived GBM cells. Cells were cultured in the brain cancer chip for 7 days, where they formed 3D spheroids. On day 7, BAY 11-7082 and TMZ were introduced in combination to the cancer spheroids by the right and left inlets, respectively, as described in the Material and Methods. Fig. 2(c) illustrates the effect of both drugs on LN229 spheroids cells over the course of 7 days after the drug administration. By day 4, the effect of the drug treatment was observed. In columns B – E, spheroids began to disrupt and collapse, corresponding to cell death. On day 7 after drug administration, spheroids in columns A, F, and G also collapsed, while the spheroids in columns B – E were completely disrupted.

**Figure 3.**
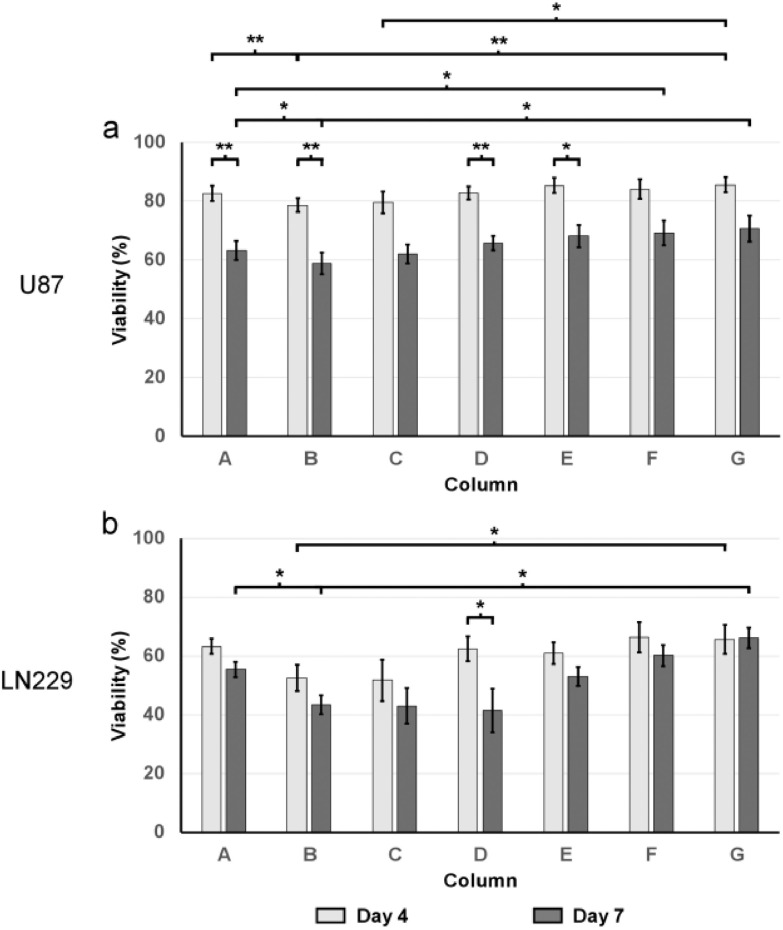
Viability of LN229 and U87 GBM cells after the drug treatment (n = 5). (a) Viability of U87 cells (b) Viability of LN229 cells. Light grey and dark grey bars represent the cell viability at Day 4 and Day 7 after the drug treatment, respectively. The white bar represents the average viability of cell spheroids before the drug was applied. All values are shown as mean ± standard error of the mean. * indicates p < 0.05, ** indicates p < 0.01.

The cell viability assay was performed before the drugs were introduced, as well as on day 4 and day 7 after drug administration. Quantitative analysis of GBM cells viability revealed differing responses within different GBM cell types and drug effectiveness over time. As shown in Fig. 3(a), the drug treatment resulted in a general decrease in viability on U87 cells. On day 4 and day 7, the average viability among all columns were 82.7 ± 2.5% and 65.4 ± 3.6%, respectively for U87 cells, while their viability was 89.1 ± 2.0% before the drug administration. Compared to U87 spheroids, the drugs were more effective on LN229 spheroids. On day 4 and day 7, the average cell viability among all columns were 60.4 ± 4.6% and 51.8 ± 4.2%, respectively. The viability of LN229 cells before drug administration was 85.1 ± 3.4% (Fig. 3(b)).

The cell viability assay also suggested the effectiveness of the NF-κB inhibitor and TMZ in combination. As shown in Fig. 3(a), on day 4 of U87 spheroids, column B showed highest drug effectiveness of 78.6 ± 2.3%, significantly (p < 0.01) lower than 82.6  ±  2.5% in channel A (TMZ side) and 85.6 ± 2.5% in channel G (BAY 11-7082 side). The LN229 spheroids on day 4 showed prominent drug response in channels B-C, with 52.6  ±  4.4% and 51.7  ±  7.0% viability respectively, compared to 63.3 ± 2.6% in channel A (TMZ side) and 65.5 ± 5.0% in channel G (BAY 11-7082 side). The day 7 data showed a more exaggerated trend that the combinational effectiveness between the BAY 11-7082 and TMZ is higher than using either of them alone. On day 7 of the U87 chip, column B revealed the highest drug effectiveness of 58.9  ±  3.7%, significantly (p < 0.05) lower than 63.3  ±  3.1% in channel A (TMZ side) and 70.6  ±  4.4% in channel G (BAY 11-7082 side). LN229 spheroids showed a similar trend (Fig. 3(b)). On day 7, LN229 spheroids showed similar high drug response in channels B-D, with 43.4  ±  3.2%, 43.0  ±  6.1%, and 41.4  ±  7.4% viability respectively, compared to 55.4  ±  2.5% in channel A (TMZ side) and 66.1  ±  3.5% in channel G (BAY 11-7082 side).

**Figure 4.**
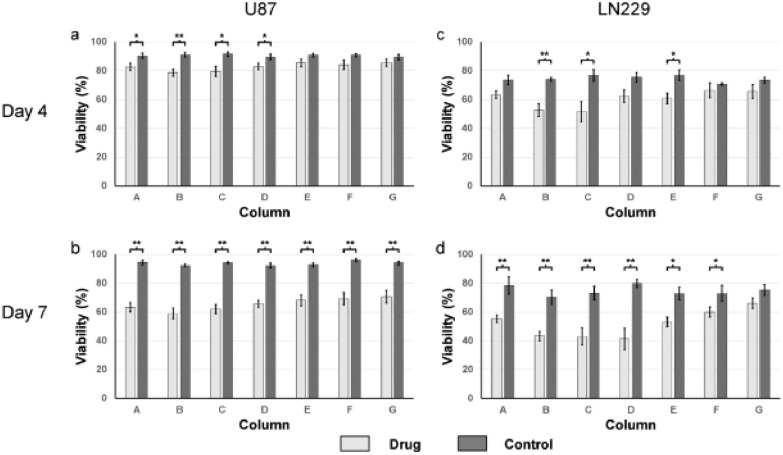
Viability of U87 and LN 229 GBM cells after drug treatment in brain chips (n = 5). Light grey and dark grey bars represent the cell viability of the drug treated cells and their respective controls at a given column, respectively. All values are shown as mean ± standard error of the mean. * indicates p < 0.05, ** indicates p < 0.01. (a) Viability of U87 cells, four days after drug treatment. (b) Viability of U87 cells, seven days after drug treatment. (c) Viability of LN229 cells, four days after drug treatment. (d) Viability of LN229 cells, seven days after drug treatment.

The cell viability for the drug-treated spheroids were significantly lower than their respective control (non-drug treated). In the controls, spheroids from different columns shared similar viability. On day 4, the negative control for the U87 and LN229 spheroids from all columns ranged from 89.4% to 91.5%, and 70.7% to 76.8%, respectively, while the viability of their counterparts that have been drug-treated are lower, ranged from 78.6% to 85.6%, and 51.7% to 66.4%, respectively (Fig. 4(a) and c). This observation was more promoted on day 7, where viability of the cells from each column for U87 cells, and from six in seven columns for LN229 cells were significantly lower than their respective controls. In details, the controls for the U87 and LN229 spheroids from all columns ranged from 92.0% to 96.0%, and 70.3% to 79.9%, respectively, while the viability of their counterparts that have been drug-treated ranged from 58.9% to 70.6%, and 41.4% to 66.1%, respectively (Fig. 4(b) and d).

**Figure 5.**
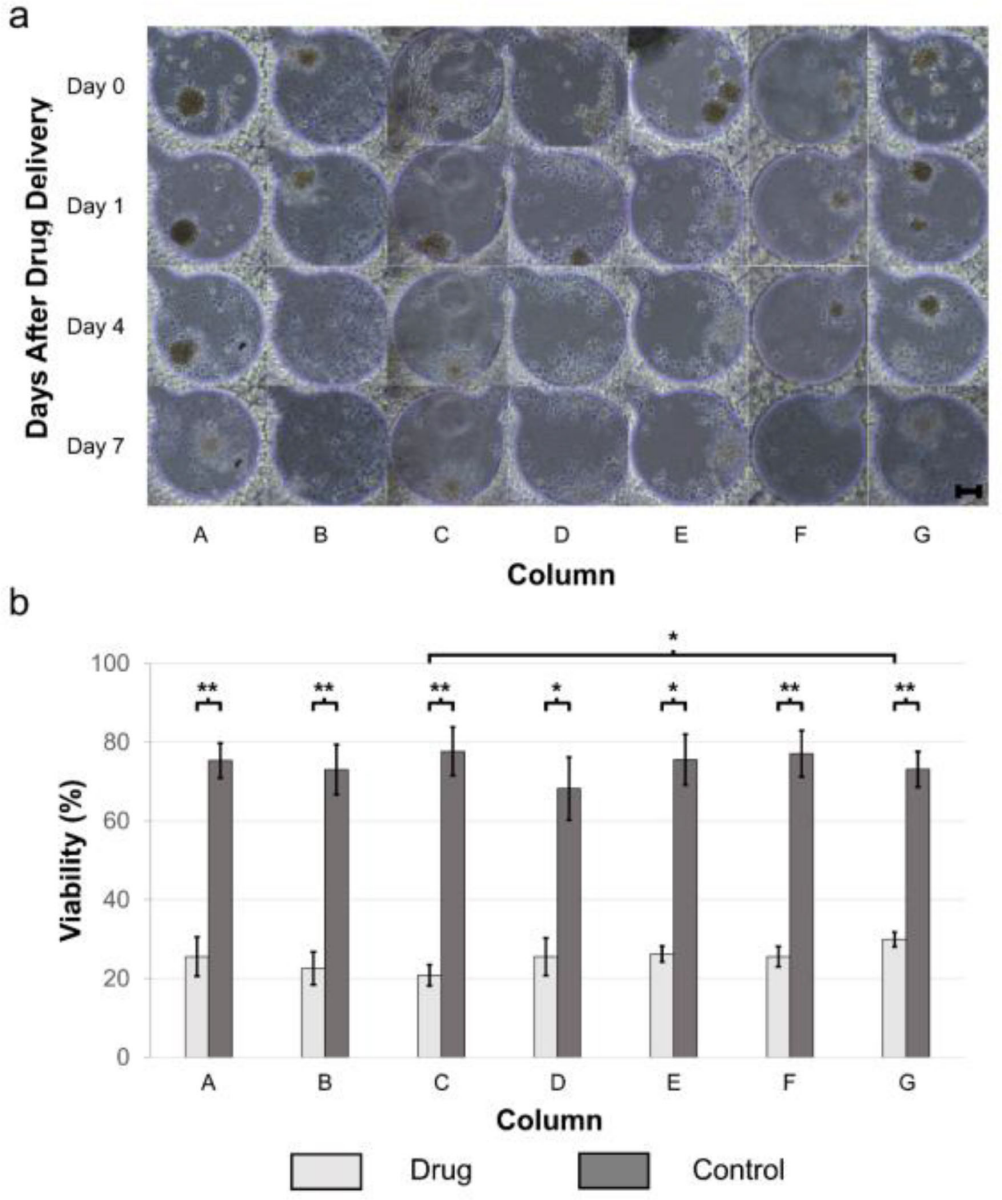
Patient-derived GBM spheroids over 7 days of culture after the drug administration. TMZ (600 μM, left inlet) and BAY 11-7082 (10 μM, right inlet) were applied to the spheroids in brain cancer chip. (a) Representative images of patient-derived GBM spheroids. Effects of the drug treatment on the GBM spheroids were visualized by the spheroid collapsing and the disaggregation of dead cells from the spheroids. Scale bar is 100 μm. (b) Viability of primary GBM cells 7 days after drug treatment (n = 5) versus negative control (n = 3). * indicates p < 0.05, ** indicates p < 0.01.

We further tested BAY 11-7082 and TMZ on patient-derived GBM tumor spheroids cultured in the brain cancer chip. Similar to what we have observed in drug treatment of GBM cell lines, we noticed an increase in the drug effectiveness over time. As demonstrated in Fig. 5(a), after the drug administration, cancer spheroids started to disaggregate dead cells (appeared on day 1 for cells in columns B-E, and on day 4 for cells in columns A, F, and G), followed by the total disruption of the spheroids (appeared on day 4 for cells in columns B-E, and on day 7 for cells in columns A, F, and G). Additionally, the cell viability assay also suggested the combinational effectiveness between the BAY 11-7082 and TMZ. As shown in Fig. 5(b), on day 7 of the patient-derived GMB spheroids, column C had the highest drug effectiveness with 15.0 ± 4.7% cell viability, compared to 20.8 ± 2.1% in channel A (TMZ side) and 25.1 ± 2.3% in channel G (BAY 11-7082 side). In contrast, the average viability of the cell spheroids in the control group was 70.5%.

## III. Discussion

In this paper, we improved a microfluidic brain cancer chip previously developed in our lab [28], [29] and demonstrated its effectiveness in culturing human GBM cells as cancer spheroids, and its ability to be used to screen GBM drugs. We implemented a thin laminar flow distribution layer in the device above the hydrogel-based microchannels, which was deep enough to allow gradient-originated distribution of single cells and the drug of interest, as shown in Fig. 2(a)-b. Once the spheroids formed in the microwells, the shallowness of this layer prevented the spheroids, given their larger diameter, from escaping. As a result, this laminar flow layer architecture increased cell capture efficiency during the initial seeding stage. Further, this *in vitro* 3D cell culture platform can reduce the time and cost of preclinical studies, and can act as a 3D *in vitro* drug screening tool.

This brain cancer chip has several important advantages over alternative hydrogel-based microfluidic methods currently used for spheroid culturing and drug screening. Simple microwell-arrays are typically fabricated with microwells in an array, then cells are directly seeded onto the array, [35]–[39] removing the effect of flow experienced by GBM in vivo. Hydrogel-based microwells or microstructure arrays enhanced with PDMS-based guide microfluidic channels, which may appear superficially similar to our brain cancer chip, have been used to capture or localize cells for spheroid culturing. Such devices are typically fabricated by non-adherent hydrogels, thus promoting the formation of spheroids, and facilitate exchange of nutrients and waste for spheroids due to the hydrogel's selective permeability properties [40], [41]. The microfluidic channels provide excellent matter exchange, making them particularly well suited for long-term spheroid culturing. These devices are not, however, suitable for practical drug screening. It is hard to perform simultaneous drug tests with various combinations and concentrations of drugs in a single microwell array due to the lack of a suitable microchannel network designed for drug distribution in gradients. Moreover, their complex fabrication process (i.e., these devices are composed by PDMS parts and hydrogel parts) limits the possibility of massive fabrication and commercialization.

In our brain cancer chip, we cultured multiple GBM cell lines as well as patient-derived GBM cells as 3D cancer spheroids. We also assessed the response of the cells to varying concentrations of two different GBM drugs, NF-κB inhibitor BAY 11-7082, and a clinical cancer drug TMZ. In the standard treatment of GBM, TMZ chemotherapy is combined with surgery and radiotherapy. Oral administration of TMZ is known as the most effective strategy for GBM. However, tumor recurrence and intrinsic or acquired TMZ resistance cause a lack of response to TMZ treatment [42]. Therefore, there is a need for a better understanding on the mechanism of TMZ resistance, and how the TMZ interact with other potential GBM drugs. Activation of NF-κB is often observed in various types of cancer including GBM and has been associated with tumor development, progression, invasion, metastasis, and chemoresistance [21], [43]–[45]. Studies have reported that NF-κB activation in GBM is also associated with the grade of the tumor, a poor prognosis, and drug resistance, specifically TMZ resistance [46], [47]. Therefore, targeting NF-κB activation or function is considered a promising strategy for inhibiting tumor growth and metastasis and for increasing therapeutic efficiency.

Compared with previous studies that have investigated the cell toxicity of TMZ on 2D GBM cell lines, 3D tumor cultures demonstrate more in vivo -like intrinsic properties and a higher survival rate under similar TMZ concentration and treatment time [48], [49]. Our results revealed differing drug responses between GBM cell lines and the GBM patient-derived cells. We observed an increase in cell death when drugs were used in combination, and a stronger effect of TMZ compared to the NF-κB inhibitor BAY 11-7082. When the cell viabilities were assessed on day 7 after the drug treatment, the highest cell deaths were observed in column B, column D, column C for U87, LN229 and patient-derived cells, respectively. It should be noted that having the increased cell death specifically in columns C and D suggests that the cells responded more to the combination of two drugs rather than TMZ or BAY 11-7082 alone. Further, our results suggested a synergistic effect of the drugs, as the effects of two different treatments were more enhanced than the sum of individual effect of the drugs alone. Provided that the cell viability decreased with the dual drug treatment, the inhibition of NF-κB combined with TMZ could possibly offer benefits for GBM patients.

Although each cell type had a slightly different response, this difference within patients was unsurprising as patient-to-patient variability in glioblastoma treatment has been well documented [31], [50]–[52]. This variability in patient response to treatment is a key factor limiting the ability to uniformly treat GBM, and emphasizes the need for a platform to provide patient-specific assessment of drug treatment options. Based on our encouraging preliminary data, we believe that our brain cancer chip could fill this role in the treatment of GBM.

Taken together, our results suggest that improved brain cancer chip enables us to simultaneous test a range of concentrations of different drugs distributed among the microwells, without using labor-intensive fabrication processes and excessively high driving pressure. Brain cancer chips minimize cell loss during the seeding stage and enable long-term tumor spheroid culture. Our findings also showed that inhibition of NF-κB when applied with TMZ plays an important role in GBM proliferation, and could provide a basis for developing new cancer therapies. Further development of this technology could enable a large-scale approach for personalized tumor treatment methods.

## IV. Materials and Methods

Please refer to the Supplementary Materials for the details on material and methods, including fabrication and improvement of the brain cancer chip devices, finite element simulation, cell lines and cell culture, cell seeding, drug administration, quantification of cell viability, and statistical analysis.

## V. Conclusions

In this work, we improved our brain cancer chip system by adding an additional laminar flow distribution layer, which reduces sample loss during cell seeding and prevents spheroids from escaping from the microwells. Using the improved brain-chip platform, we could optimize the use of tumor samples derived from GBM patients to provide valuable insight on the tumor growth and responses to drug therapies. We cultured 3D spheroids from GBM cell lines and patient-derived GBM cells in vitro, and investigated the effect of the combination of Temozolomide and nuclear factor-κB inhibitor on tumor growth. Our study revealed that these drugs have synergistic effects in inhibiting spheroid formation when used in combination. These results suggest that the brain cancer chip enables large-scale, inexpensive and sample-effective drug screening to 3D cancer tumors in vitro, and could be applied to related tissue engineering drug screening studies.

## Supplementary Materials

The supplementary materials include the fabrication of improved brain chip, the finite element simulation, the details of cell lines and cell culture, the quantification of cell viability and the statistical analysis.


