# Supplementary material for: Temozolomide in Combination With NF-κB Inhibitor Significantly Disrupts the Glioblastoma Multiforme Spheroid Formation
Source: IEEE Open J Eng Med Biol. 2020 Feb 17;1:9–16. doi: 10.1109/OJEMB.2019.2962801 (PMC8983150; doi:10.1109/OJEMB.2019.2962801)
Supplement: Supplementary file 1 [file supp1-2962801.pdf]

Supplementary Materials

Temozolomide in Combination with NF-κB Inhibitor Significantly Disrupts the Glioblastoma Multiforme Spheroid Formation

Hui Xia, Naze G. Avci, Yasemin Akay, *Member, IEEE*, Yoshua Esquenazi Levy, Lisa H. Schmitt, Nitin Tandon, Jay-Jiguang Zhu, and Metin Akay*, *Fellow, IEEE*

THE fabrication process of the brain cancer chip has been previously described [29]. Briefly, cover glass slides were treated with 3-(Trimethoxysilyl)propyl methacrylate 98% (TMSPMA) to generate an adhesive surface suitable for crosslinking to the PEGDA hydrogel. A glass coverslip slide was prepared using a 100 W CO_2 laser cutter (CAMFive). Two inlets, a single outlet, and six pairs of vent holes for uncross-linked hydrogel removal were cut into the top glass slide, and a filling port was cut into the bottom slide. Reservoirs were affixed using epoxy glue (Loctite, Hartford, CT) to the inlets and outlets to provide space for phosphate buffered saline (PBS) or culture media to be loaded. An acrylic frame 500 µm in thickness was cut to shape using the same CO_2 laser, and the frame was placed between two treated cover glass slides. The liquid hydrogel solution was prepared by dissolving monomeric PEGDA (20% v/v, Polysciences, Inc., Warrington, PA) and a photoinitiator, 2-Hydroxy-4-(2-hydroxyethoxy)−2 methylpropiophenone (PI, Sigma Aldrich, St. Louis, MO) in Dulbecco's (D)-PBS. 1 mL of the solution was loaded into the chip frame through the filling port, and a photomask was aligned to the inlets, outlet, and the holes cut for the diffusion prevention gaps. The photomasks were designed using AutoCAD (AutoDesk, Inc., CA) and printed onto a plastic surface with a high level of transparency (CADart, Bandon, OR). Photo-polymerization occurred via UV exposure with an Omnicure S2000 (320–500 nm, EXFO, Ontario, Canada) lamp at 20 mW/cm^2 (365 nm) at a distance of 11 cm for a given time period. The chip was then placed in a 2.5-inch petridish (VWR, Radnor, PA). The polymer solution that was not yet crosslinked was removed by loading 1 mL of sterile PBS at each inlet, and allowed to perfuse through the microchannel. After three PBS washes, the petridish was placed upside-down and exposed to flooding UV for 30 min, then placed upright again and exposed to flooding UV for another 15 min. This process was carried out to crosslink any remaining unlinked hydrogel solution, while sterilizing the chip. The fabricated chips were stored in PBS at room temperature until use.

A. *Improvement of brain cancer chip devices*

Compared with the often-used microfabrication materials, such as silicon, glass, and epoxy photoresist, the crosslinked PEGDA hydrogel is relatively rough, as shown in Fig. 2 and Fig. 5. As a result, it is hard to measure the depth of the laminar flow layer directly with conventional equipment such as ellipsometer or profilometer. To estimate the depth of the laminar flow layer, we measured the flow perfusion rate of the chip. The chip was tilted at 45 degrees, and perfused with PBS in the inlets. During the measurement, the inlets were kept filled to ensure the pressure drop from the inlet to the outlet remained consistent. The fluid from the outlet was collected and weighed. The decrease in the pressure of the chip (ΔP) could be derived using the density of PBS (ρ), gravity acceleration (g), and the height the fluid dropped form the inlets to the outlet (Δh) using Pascal's law:

$$\Delta P = \rho g \Delta h \qquad (1)$$

The pressure drop of the chip can also be expressed using the flow perfusion rate (Q) and the total flow resistance of the chip (R_t), using an alternation of Poiseuille's law:

$$\Delta P = QR_t \qquad (2)$$

Using (1) and (2), one can derive the total flow resistance of the chip from measured pressure drop and perfusion rate. On the other hand, as the brain chip was composed of microwell arrays that were connected with microchannels of various length and width, the fluidic resistance of the entire channel network (R_c) could be broken up into individual microchannels. The flow resistance of each channel can be estimated by approximating these microchannels as rectangular shape. The fluidic resistance of a rectangular channel is given by (3), in which L is the length, w is the width, d is the depth of the microchannel, and μ is the dynamic viscosity of the fluid [53, 54].

$$R = \frac{12\mu L}{dw^3}\left[\sum_{n=1,3,5\ldots}^{\infty} \frac{\tanh\left(\frac{n\pi d}{2w}\right)}{(n\pi)^3}\right]^{-1} \qquad (3)$$

Using (3), the microfluidic network was converted to a grid of flow resistors. Making an analogy to electrical circuits, the fluidic resistance of the entire channel network (R_c) was then calculated from the resistances of individual microchannels using the Kirchhoff's Voltage Law. Considering that the flow resistance of the microfluidic channel network (R_c) and the flow resistance of the laminar flow layer (R_l) could be considered as parallel resistors, one can derive R_l using (4) as Rc and the total flow resistance of the chip (R_t) are known:
Placing R_l back into (3), one will be able to derive the depth of the laminar flow layer, as the length and width of the laminar flow layer is known.

$$R_l = \frac{R_t R_c}{R_c\text{-}R_t} \qquad (4)$$

B. *Finite element simulation*

The original and the laminar flow layer enhanced brain chips were modelled using COMSOL Multiphysics (www.comsol.com) to calculate drug concentration in each of the microchannels. Two 3D models were implemented using the geometry of each of the brain chips. A 600 µM TMZ solution and a 10 µM BAY 11-7082 solution were injected into the left and right inlets, respectively. Assuming creeping flow and a stationary solver, the fluid concentration rates in the microwells along the channels were estimated using the incompressible Stokes equations.

C. *Cell lines and cell culture*

Glioblastoma cell lines LN229 and U87 were purchased from the American Tissue Culture Collection (ATCC) (USA). GBM cells were grown in Dulbecco's modified Eagle's medium (DMEM) (Gibco, USA) supplemented with 10% FBS (HyClone, USA), 100 U/mL penicillin (Gibco, USA), and 100 g/mL streptomycin (Gibco, USA). We obtained resected glioblastoma tumors from the UTHealth and Memorial Hermann, Texas Medical Center, Houston, Texas. All the experimental protocols were approved by the committee for the Protection of Human Subjects at UTHealth and University of Houston, and informed consent for participation in this study was obtained from each subject. All methods were performed in accordance with the relevant guidelines [29]. The patient-derived GBM tumor cells were placed in Neurobasal Medium (StemCell Technologies) supplemented with B27, N2, glutamax, heparin sulphate, hFGF and EGF. All cells were stored in a cell culture incubator with 5% CO_2 at 37 °C.

D. *Cell seeding*

The cells in their respective medium were cultured at a concentration of 1 x 10^6 cells/mL. Each inlet of the chip was loaded with 0.25 mL of the cell dilute. To measure the percentage cell loss (as shown in Fig. 1d), LN229 cells in DMEM at concentration of 1 x 10^6 cells/mL were loaded into the chip. After the chip was stationary for 15 minutes, the fluid at the inlets and the outlet were sampled and subjected to cell counting. The percentage cell loss is calculated as below in (5):

$$\text{Cell loss} = \frac{\text{cell concentration at the outlet}}{\text{cell concentration at the inlet}} \qquad (5)$$

E. *Drug administration*

After 7 days of spheroid culture in the brain cancer chip, a combination of drugs was applied to the chip. TMZ (Sigma Aldrich, St. Louis, MO) was dissolved in dimethysulfoxide (DMSO) to prepare a solution of 10 mM TMZ. The solution was diluted to 600 µM TMZ in Nerobasal media. Cell culture media was removed from the chip and replaced by adding 600 µM TMZ to the right inlet and 10 µM NF-κB to the left inlet [32, 55]. Drug administration was done only once, and cells were left in the brain cancer chip for 7 days following drug administration. Control (non-drug treated) brain cancer chips were maintained under the same conditions and were established using same cells as the drug-treated sample.

F. *Quantification of cell viability*

In order to quantify the viability of the spheroids after drug administration, the spheroids were removed from the chip and washed twice with PBS, then digested with trypsin for cell dissociation. Viability was quantitatively examined following staining with 0.4% trypan blue solution and counting using a haemocytometer.

G. *Statistical analysis*

Statistical analysis was calculated using Student's t-test. Confidence intervals were set at 95% ($p < 0.05$) and 99% ($p < 0.01$). Error bars are mean ± standard error.
